# Elevated triglyceride-glucose index is a risk factor for progression to prehypertension in normoglycemic Japanese: a 5-year retrospective cohort study

**DOI:** 10.1186/s40885-024-00293-6

**Published:** 2024-12-01

**Authors:** Masanori Shimodaira, Yu Minemura, Tomohiro Nakayama

**Affiliations:** 1 Department of Internal Medicine, Takara Clinic, Nagano, Japan; 2 Division of Laboratory Medicine, Department of Pathology and Microbiology, Nihon University School of Medicine, Tokyo, Japan

**Keywords:** Glucose, Prehypertension, Risk factors, Triglycerides

## Abstract

**Background:**

The triglyceride**-**glucose (TyG) index is an alternative biomarker of insulin resistance that may be associated with elevated blood pressure. However, the relationship between the TyG index and the risk of prehypertension remains unclear. This longitudinal, retrospective cohort study aimed to investigate the connection between the TyG index and the risk among Japanese population.

**Methods:**

We enrolled 17,758 participants who underwent medical health checkups in 2017 (baseline) and 2022. At baseline, all participants were normotensive and normoglycemic state, and none were using triglyceride-lowering medications. Participants were divided into four groups according to quartiles of the TyG index at baseline. The risk of progressing to prehypertension was evaluated using multivariable Cox proportional hazard models. In addition, multivariate restricted cubic spline analysis was conducted to examine the dose–response relationship. Furthermore, receiver operating characteristic (ROC) curve analysis was performed to determine the predictive value of the TyG index for progression to prehypertension.

**Results:**

Compared with the lowest quartile (Q1) of the TyG index group, the adjusted hazard ratios (95% confidence intervals) for progression to prehypertension in the Q2, Q3, and Q4 groups were 1.05 (0.95–1.19), 1.14 (1.02–1.30), and 1.28 (1.11–1.50), respectively. The restricted cubic spline analysis demonstrated a dose–response relationship between the TyG index and the risk of prehypertension. The area under the ROC curve was 0.60 (0.59–0.61), demonstrating a sensitivity of 56.2% and specificity of 58.8%.

**Conclusions:**

The findings suggest that an elevated TyG index may be independently and positively associated with an increased risk of progression to prehypertension in the Japanese population.

## Background

Hypertension (HTN) is a crucial risk factor for cardiovascular diseases and is widely prevalent worldwide. Prehypertension (preHTN) is defined as a systolic blood pressure (SBP) of 120 to 139 mmHg and/or diastolic BP (DBP) of 80 to 89 mmHg [1], affecting 25% to 50% of adults worldwide [2]. Numerous prospective cohort studies and meta-analyses have consistently demonstrated that individuals diagnosed with preHTN face a twofold to threefold higher risk of progressing to HTN [3]. Moreover, HTN and preHTN are associated with high cardiovascular disease mortality rates compared with BP levels < 120/80 mmHg [4]. Therefore, identifying individuals at risk of progressing to preHTN could help prevent morbidity and mortality associated with HTN-related diseases.

The pathogenesis of preHTN presents a multifaceted challenge; however, its underlying mechanism is still not fully elucidated. Insulin resistance is recognized as a significant contributor to HTN development [5]. Although the hyperinsulinemic-euglycemic clamp technique remains the gold standard for assessing insulin resistance, its complexity and time-intensive characteristic limit its applicability in routine clinical practice or large-scale cohort studies. Recently, the triglyceride (TG)-glucose (TyG) index has garnered attention as a reliable surrogate index of insulin resistance that does not require insulin measurement. The TyG index, which is calculated as “Ln [TG (mg/dL) × fasting plasma glucose (mg/dL) / 2],” showed the highest sensitivity (96.5%) and specificity (85.0%) in predicting insulin resistance assessed using the hyperinsulinemic-euglycemic clamp technique [6].

Given the established connection between insulin resistance and high BP and the reflective nature of the TyG index on insulin resistance, TyG may be linked with high BP. Notably, a community-based survey of a Chinese cohort revealed that the odds ratio for preHTN was upregulated across TyG index quartiles, up to 2.06 (95% confidence interval [CI], 1.53–2.77) for the highest quartile versus the lowest quartile [7]. Similarly, some epidemiologic studies have also supported the positive association between a high TyG index and preHTN [8–11]; however, studies have reported conflicting results [12, 13]. Results of most studies were limited to populations, such as older adults [7, 10], children and adolescents [8], lean adult subjects [13], or individuals with normoglycemia defined only by fasting blood glucose (FPG) levels without hemoglobin A1c (HbA1c) [12]. Furthermore, these studies were conducted as case–control or cross-sectional investigations [7–13], and the longitudinal association between the TyG index and progression to preHTN remains unclear. Therefore, this 5-year retrospective, longitudinal cohort study was conducted to identify the causal association in normoglycemic Japanese individuals defined by FPG and HbA1c levels.

## Methods

### Study design and participants

**Fig. 1:**
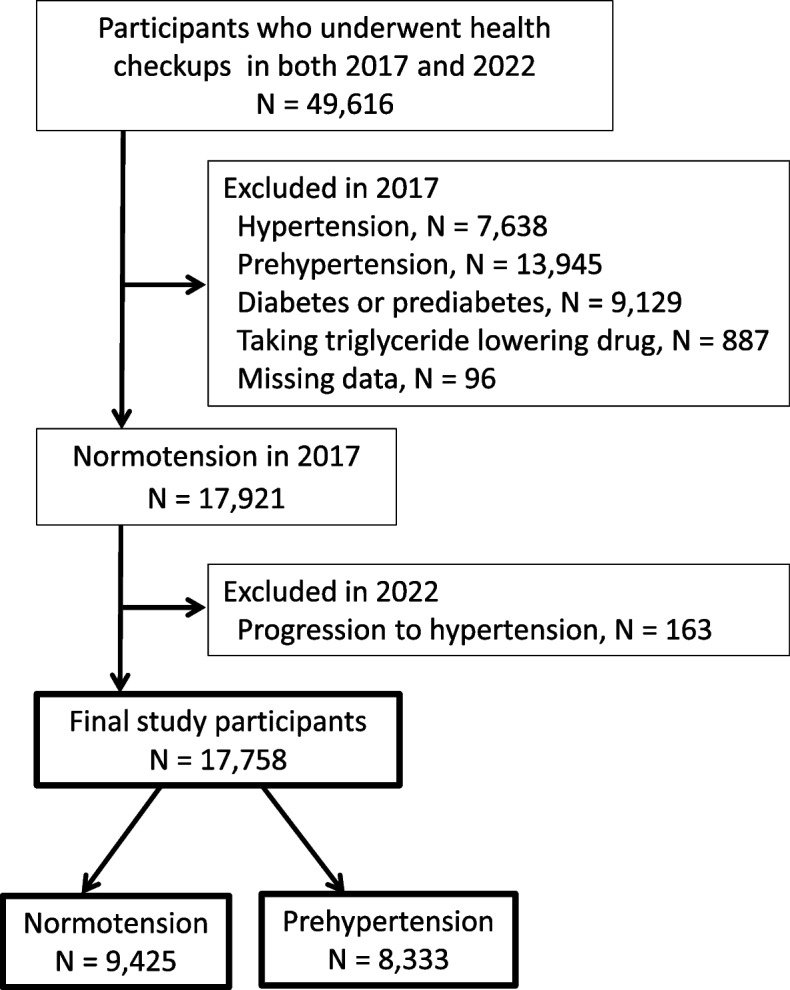
Flowchart of the screening process for the selection of eligible participants

This cohort study was conducted in a rural city located in Nagano Prefecture, Japan, and analyzed data from the Chubu Public Health Center. Initially, Japanese participants who underwent annual health checkups at the center in 2017 (baseline) were examined, and their health status was reassessed 5 years later. Subsequently, participants with HTN (*n* = 7,638), preHTN (*n* = 13,945), diabetes (*n* = 1,024), prediabetes (*n* = 8,105), those taking TG-lowering agents (*n* = 887), and those with missing TG and/or FPG data were excluded (*n* = 96). From the initial cohort of 17,921 participants, 163 who were normotensive but progressed to HTN during the 5-year follow-up were further excluded. Ultimately, this study analyzed data from 17,758 participants (9,425 participants who remained normotensive and 8,333 participants who progressed to preHTN) (Fig. 1).

### Anthropometric and clinical measurements

Physical assessments were conducted to evaluate parameters including height, weight, waist circumference (WC), SBP, and DBP by proficient examiners. The participant’s BP was measured in the left arm while seated after a 5-min rest period, using an automatic BP monitor device that utilizes the cuff-oscillometric method. Body mass index (BMI) was calculated from weight (kg) divided by height squared (m^2^).

Venous blood samples were obtained after an overnight fast. Serum levels of total cholesterol (TC), low-density lipoprotein cholesterol (LDL-C), high-density lipoprotein cholesterol (HDL-C), TG, uric acid (UA), FPG, HbA1c, and creatinine were measured by standard methods using an automatic analyzer (Hitachi 47, Hitachi Ltd). The TyG index was denoted as “Ln [TG (mg/dL) × FPG (mg/dL) / 2]” [6]. The estimated glomerular filtration rate (eGFR) was calculated using the formula recommended by the Japanese Society of Nephrology: “194 × serum creatinine^−1.094^ × age^−0.287^ mL/min/1.73 m^2^,” with an additional multiplication factor of 0.739 for female participants [14].

### Definition criteria

In accordance with the definitions provided by the American Diabetes Association, diabetes was defined as FPG of ≥ 126 mg/dL and HbA1c of ≥ 6.5%, or the use of antidiabetic medications. Prediabetes was defined as FPG of 100 to 125 mg/dL, or HbA1c of 5.7% to 6.4%, whereas normoglycemia was defined as FPG of < 100 mg/dL and HbA1c of < 5.7% [15].

HTN was defined as SBP ≥ 140 mmHg or DBP ≥ 90 mmHg, or the use of any antihypertensive medications. PreHTN status was defined as SBP of 120 to 139 mmHg or DBP of 80 to 89 mmHg, without taking an antihypertensive medication. Normotensive participants were defined as those with SBP < 120 mmHg and DBP < 80 mmHg [1].

In accordance with criteria established in Japan, obesity was defined as a BMI of ≥ 25 kg/m^2^ [16]. Smoking status was categorized into current smoking (daily and occasional smoking) and nonsmoking (never and former smoking). Alcohol consumption was classified as current drinkers (alcohol consumption at least once per week) and noncurrent drinkers. A familial history of HTN was identified as having one or more relatives (parent or sibling) with HTN. Medical history, smoking and alcohol status, medication usage (e.g., antidiabetic, antihypertensive, and TG-lowering medications), and familial history of HTN were collected using a structured questionnaire.

### Statistical analysis

The baseline characteristics of the study participants were classified based on quartile groups according to the TyG index. Normally distributed variables were presented as mean ± standard deviation, whereas skewed distributions of TG were depicted using the median and interquartile range. Categorical data were represented as percentages. Quartile groups were compared using the analysis of variance for continuous data, the Kruskal–Wallis test for skewed continuous data, and the chi-square test for categorical data.

The Cox proportional hazards model was utilized to investigate the association between the TyG index and the risk of progression to preHTN. With the lowest quartile group (Q1) of the TyG index as the reference, the hazard ratio (HR) and 95% CI were calculated to assess the relationship between the TyG index and the progression risk. To evaluate the potential for effect modification of TyG index quartiles and the progression risk, the analyses were stratified by sex, age (< 45 and ≥ 45 years), and BMI (< 25 and ≥ 25 kg/m^2^). In addition, restricted cubic splines with three knots representing TyG index quartiles were constructed to investigate potential nonlinear associations between the TyG index and the risk of progression to preHTN while controlling for confounding factors. Finally, the receiver operating characteristic (ROC) curve and area under the curve (AUC) were derived to evaluate the discriminatory power and utility in identifying the progression to preHTN.

All statistical analyses were performed using IBM SPSS ver. 21.0 (IBM Corp) and R ver. 4.2.3 (R Foundation for Statistical Computing). The probability of < 0.05 (two-sided) was considered a statistically significant level.

## Results

### Baseline characteristics

This study included 17,758 participants, with an average of 43.2 years, and 52.4% were male. Table 1 presents the baseline characteristics of the participants divided into quartiles based on the TyG index (Q1, < 7.64; Q2, 7.64–7.98; Q3, 7.99–8.37; Q4, > 8.38). Participants in higher quartiles tended to be male and older, with high BMI, WC, SBP, DBP, TC, LDL-C, TG, UA, and FPG values but lower HDL-C and eGFR.

**Table 1:** Baseline characteristics of participants grouped by TyG index quartiles

Variable	TyG index				P for trend
	Q1 (*n* = 4,429)	Q2 (*n* = 4,441)	Q3 (*n* = 4,445)	Q4 (*n* = 4,443)	
TyG					
Range	< 7.64	7.64–7.98	7.99–8.37	> 8.38	< 0.001
Average	7.4 ± 0.2	7.8 ± 0.1	8.2 ± 0.1	8.8 ± 0.4	< 0.001
Male sex (%)	32.5	41.1	51.3	66.4	
Age (yr)	37.1 ± 12.2	41.2 ± 13.7	43.9 ± 14.5	45.5 ± 14.5	< 0.001
Body mass index (kg/m^2^)	20.2 ± 2.6	20.7 ± 2.8	21.5 ± 3.0	22.8 ± 3.3	< 0.001
Waist circumference (cm)	72.0 ± 6.9	74.2 ± 7.6	76.8 ± 8.1	81.4 ± 8.8	< 0.001
Systolic blood pressure (mmHg)	105.8 ± 8.5	106.9 ± 8.2	108.2 ± 7.8	109.3 ± 7.6	< 0.001
Diastolic blood pressure (mmHg)	64.7 ± 7.0	65.9 ± 6.9	66.9 ± 6.8	67.9 ± 6.9	< 0.001
Total cholesterol (mg/dL)	185.8 ± 31.3	194.5 ± 31.9	201.7 ± 32.4	213.4 ± 36.0	< 0.001
LDL-C (mg/dL)	102.5 ± 25.3	112.8 ± 26.3	121.4 ± 28.9	131.3 ± 32.1	< 0.001
HDL-C (mg/dL)	69.9 ± 14.3	66.9 ± 13.8	62.7 ± 13.7	54.7 ± 13.2	< 0.001
Triglyceride (mg/dL)	38.7 (14–72)	57.2 (29–104)	79.2 (42–156)	147.8 (60–270)	< 0.001
Uric acid (mg/dL)	3.5 ± 2.4	3.7 ± 2.4	4.1 ± 2.5	4.3 ± 2.7	< 0.001
eGFR (mL/min/1.73 m^2^)	81.9 ± 14.3	78.8 ± 14.5	76.9 ± 14.9	78.2 ± 14.8	< 0.001
Fasting plasma glucose (mg/dL)	84.4 ± 7.1	87.5 ± 7.5	89.9 ± 8.5	94.2 ± 14.0	0.003
Hemoglobin A1c (%)	5.3 ± 0.2	5.3 ± 0.2	5.4 ± 0.3	5.4 ± 0.4	0.070
Current drinker (%)	32.3	34.6	35.7	37.2	< 0.001
Current smoker (%)	17.2	21.2	25.4	36.2	< 0.001
Family history of hypertension (%)	13.9	14.9	15.0	17.7	< 0.001

Values are presented as mean ± standard deviation, median (interquartile range), or number only, unless otherwise indicated

*TyG* Triglyceride-glucose, *Q* quartile, *LDL-C* Low-density lipoprotein cholesterol, *HDL-C* high-density lipoprotein cholesterol, *eGFR* Estimated glomerular filtration rate

### Risk of progression to preHTN according to TyG index quartiles

**Fig. 2:**
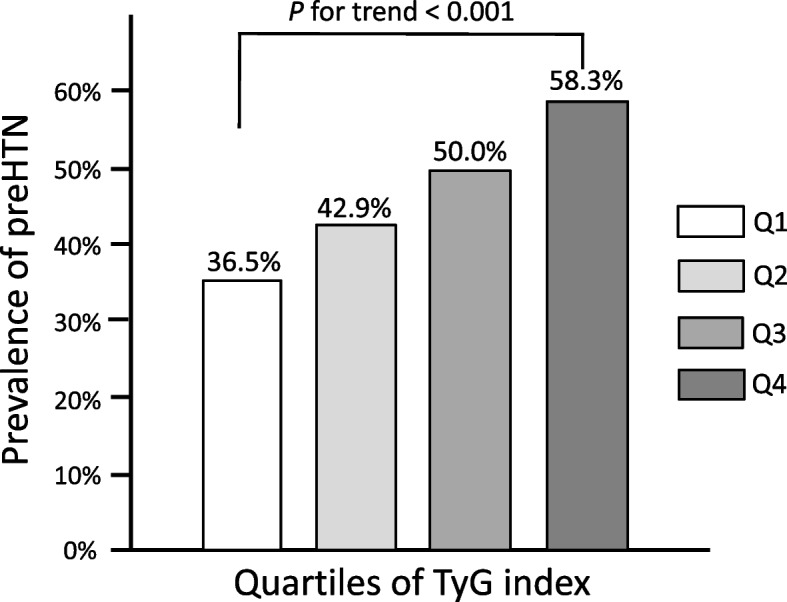
Proportion of progression to prehypertension (preHTN). TyG, triglyceride-glucose; Q, quartile

Figure 2 shows the proportion of participants progressing to preHTN according to the TyG index quartiles. The proportion exhibited an upward trend with increasing TyG index quartiles, reaching 36.5%, 42.9%, 50.0%, and 58.3% in Q1, Q2, Q3, and Q4, respectively. Table 2 presents the results of the Cox proportional hazard models assessing the HRs in each TyG index quartile. Model 1 was adjusted for sex, age, BMI, WC, SBP, and DBP at baseline. Model 2 also incorporated adjustments for HDL-C, LDL-C, UA, eGFR, and HbA1c at baseline. Model 3 further accounted for smoking and drinking status, along with the family history of HTN. In model 3, the HRs revealed a positive and independent association between the TyG index and the risk of progressing to preHTN. Compared with Q1, the HRs in Q3 and Q4 were 1.14 (95% CI, 1.02–1.30) and 1.28 (95% CI, 1.11–1.50), respectively. Furthermore, the risk of progression to preHTN increased by 14% with every 1 standard deviation increase in the TyG index, even after adjusting for confounders.

**Table 2:** The association of TyG index with the risk of progression to preHTN

TyG index	Incdence of preHTN (%)	Crude model		Model 1		Model 2		Model 3	
		HR (95% CI)	*P*-value	HR (95% CI)	*P*-value	HR (95% CI)	*P*-value	HR (95% CI)	*P*-value
Quartile									
Q1 (< 7.64)	36.5	1.00 (Reference)		1.00 (Reference)		1.00 (Reference)		1.00 (Reference)	
Q2 (7.64–7.98)	42.9	1.31 (1.20–1.42)	< 0.001	1.09 (0.99–1.22)	0.183	1.02 (0.87–1.22)	0.319	1.05 (0.95–1.19)	0.361
Q3 (7.99–8.37)	50.0	1.74 (1.60–1.90)	< 0.001	1.25 (1.11–1.40)	0.013	1.19 (1.05–1.37)	0.018	1.14 (1.02–1.30)	0.023
Q4 (> 8.38)	58.3	2.44 (2.23–2.65)	< 0.001	1.50 (1.24–1.80)	0.011	1.35 (1.19–1.56)	0.020	1.28 (1.11–1.50)	0.029
P for trend			< 0.001		< 0.001		< 0.001		< 0.001
Category									
Q1	36.5	1.00 (Reference)		1.00 (Reference)		1.00 (Reference)		1.00 (Reference)	
Q2–Q4	51.7	1.74 (1.64–1.85)	< 0.001	1.29 (1.18–1.39)	< 0.001	1.26 (1.11–1.44)	0.001	1.21 (1.05–1.39)	0.010
Continuous (per 1.0 increase in TyG index)		1.28 (1.20–1.36)	< 0.001	1.22 (1.16–1.29)	< 0.001	1.19 (1.09–1.31)	0.013	1.14 (1.04–1.26)	0.025

Model 1, adjusted for sex, age, body mass index, waist circumference, systolic blood pressure, and diastolic blood pressure at baseline. Model 2, adjusted for high-density lipoprotein cholesterol, low-density lipoprotein cholesterol, uric acid, estimated glomerular filtration rate, and hemoglobin A1c in addition to model 1. Model 3, adjusted for smoking and drinking status, and family history of hypertension in addition to model 2

*TyG* triglyceride-glucose, *preHTN* prehypertension, *HR* hazard ratio, *CI* confidence interval

### Association of TyG index quartiles with preHTN by sex, age, and BMI

The results of the stratified analyses by sex, age, and BMI are shown in Table 3. For age-stratified analyses, considering that the mean age of all participants was 43.2 years, they were stratified into two groups: individuals aged < 45 years and those ≥ 45 years. The results indicate that age significantly modified the association between the TyG index and the risk of progression to preHTN (P for interaction = 0.036). Furthermore, the association of the TyG index with the risk of progression to preHTN was more prominent in women than in men (P for interaction = 0.012). However, no interactions were found between BMI categories and the risk of progressing to preHTN.

**Table 3:** Stratified analyses of the association of TyG index and progression to preHTN

Subgroup	TyG index				P for interaction
	Q1	Q2	Q3	Q4	
Sex					0.036
Male					
Incidence of preHTN (%)	47.4	52.9	56.0	62.0	
HR (95% CI)	1.00 (Reference)	1.08 (0.93–1.22)	1.14 (1.02–1.26)	1.23 (1.11–1.40)	
Female					
Incidence of preHTN (%)	31.2	35.9	43.7	50.9	
HR (95% CI)	1.00 (Reference)	1.11 (0.94–1.19)	1.23 (1.09–1.39)	1.41 (1.26–1.71)	
Age (yr)					0.012
< 45					
Incidence of preHTN (%)	32.2	38.0	42.8	52.1	
HR (95% CI)	1.00 (Reference)	1.11 (0.93–1.28)	1.18 (1.05–1.33)	1.41 (1.24–1.59)	
≥ 45					
Incidence of preHTN (%)	46.8	49.5	57.3	63.8	
HR (95% CI)	1.00 (Reference)	1.03 (0.91–1.16)	1.11 (1.02–1.30)	1.20 (1.09–1.37)	
Body mass index (kg/m^2^)					0.605
< 25					
Incidence of preHTN (%)	35.6	41.9	48.2	54.7	
HR (95% CI)	1.00 (Reference)	1.12 (1.03–1.29)	1.15 (1.02–1.29)	1.29 (1.14–1.45)	
≥ 25					
Incidence of preHTN (%)	54.1	57.7	65.5	71.0	
HR (95% CI)	1.00 (Reference)	1.03 (0.69–1.43)	1.13 (0.98–1.29)	1.27 (1.14–1.55)	

Adjusted for sex, age, body mass index, waist circumference, systolic blood pressure, diastolic blood pressure, high-density lipoprotein cholesterol, low-density lipoprotein cholesterol, uric acid, estimated glomerular filtration rate, hemoglobin A1c, smoking and alcohol status, and family history of hypertension, except for the variable used in each stratified analysis. P for interactions were obtained from likelihood ratios

*TyG* triglyceride-glucose, *preHTN* prehypertension, *Q* quartile, *HR* hazard ratio, *CI* confidence interval

### Dose-dependent relationship with the risk of progression to preHTN

**Fig. 3:**
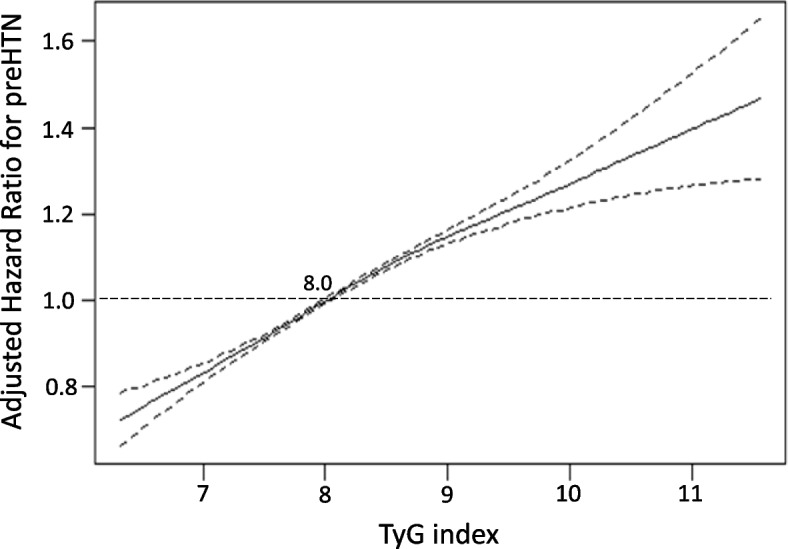
Dose–response relationship between triglyceride-glucose (TyG) index and risk of progression to prehypertension (preHTN). The restricted cubic spline analyses adjusted for confounding factors, namely, age, sex, body mass index, systolic blood pressure, diastolic blood pressure, high-density lipoprotein cholesterol, low-density lipoprotein cholesterol, uric acid, estimated glomerular filtration rate, hemoglobin A1c, smoking and drinking status, and family history of HTN. The solid line and dashed line represent the estimated values and their corresponding 95% confidence interval

Multivariable-adjusted restricted cubic spline analysis was conducted using model 3 (Fig. 3). The *P*-value for the nonlinear association was 0.37 (not significant), indicating a linear association between the TyG index and the risk of progression to preHTN. Consequently, a higher TyG index is significantly and dose-dependently associated with an increased risk of progression to preHTN.

### ROC analysis for predicting progression to preHTN

**Fig. 4:**
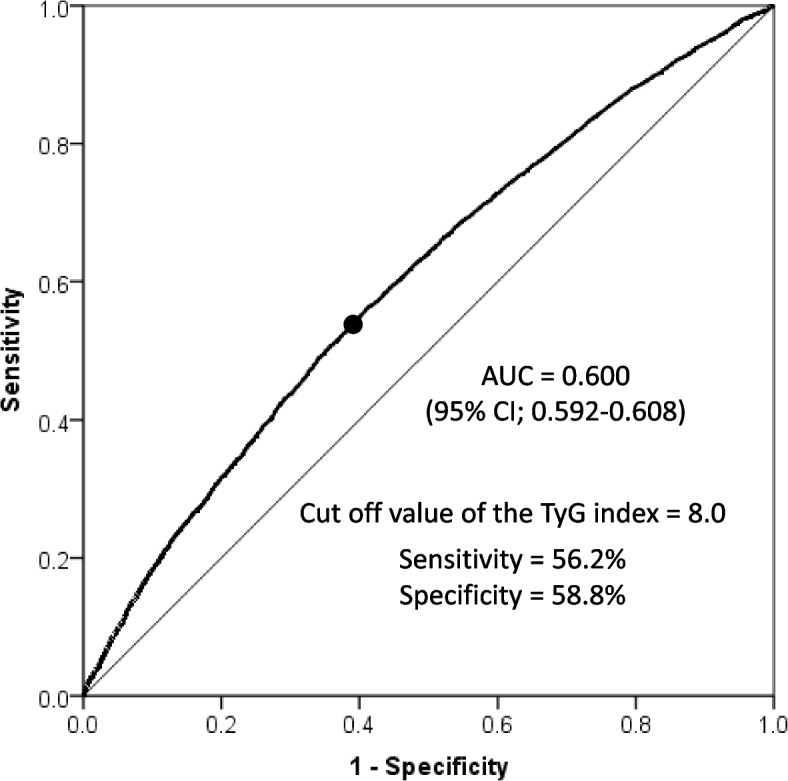
Receiver operating characteristic curve analyze of the triglyceride-glucose (TyG) index in identifying progression to prehypertension. AUC, the area under the curve; CI, confidence interval

The ROC curve analysis demonstrated a significant association between the TyG index and progression to preHTN (Fig. 4). The optimal cutoff value of the TyG index for predicting preHTN was found to be 8.0. However, the sensitivity and specificity at the optimal cutoff values were both < 0.6, indicating suboptimal performance. The model exhibited low discriminatory power (AUC < 0.7) for distinguishing progression to preHTN, with an AUC of 0.60.

## Discussion

To the best of our knowledge, this is the first retrospective, longitudinal cohort study investigating the relationship between the TyG index, a straightforward surrogate marker of insulin resistance, and the risk of progression to preHTN. Our findings reveal a noteworthy positive association between the TyG index and the risk of progression to preHTN among the Japanese population. Notably, this association remained statistically significant even after adjusting for potential confounders. Furthermore, the dose–response curve illustrates a clear positive and linear correlation between the TyG index and the risk of progression to preHTN. Consequently, this study offers novel insights for the primary prevention of preHTN.

This 5-year longitudinal observational study enrolled middle-aged participants (mean age, 43.2 years). Recently, Wang et al. [17] conducted a longitudinal study in older individuals (mean age, 60.5 years) with an observation period of 9.37 years and examined the relationship between the TyG index and HTN. Their results showed a linear dose–response relationship between the TyG index and HTN onset, with an HR of 1.21 (95% CI, 1.13–1.29) for HTN per 1-unit increase in the TyG index. Our and their findings indicate that an increase in the TyG index is associated with increased BP in middle-aged and older individuals.

Although the precise mechanisms linking the increased TyG index, which indicates worsening insulin resistance, to increased BP are not fully understood, several hypotheses have been proposed. First, insulin resistance promotes the activity of the sympathetic nervous system, which can lead to increased peripheral vascular resistance [18]. In addition, hyperinsulinemia resulting from insulin resistance helps activate the renin–angiotensin–aldosterone system, thereby promoting renal sodium retention [19]. This, in turn, can indirectly promote water–sodium retention and increase vascular activity through the action of angiotensin II [20]. Moreover, insulin resistance can impair the activation of endothelial nitric oxide synthase and hasten endothelial dysfunction, leading to systemic vasoconstriction [21]. These phenomena constitute the most prevalent theories linking insulin resistance to elevated BP.

An 8.5-year prospective study conducted in Spain reported a positive association between the TyG index and new-onset HTN in the general population [22]. Similarly, a 9-year longitudinal study of a Chinese population showed a cumulative incidence of HTN in Q1, Q2, Q3, and Q4 of the TyG index of 28.5%, 36.9%, 49.2%, and 59.8%, respectively (P for trend < 0.001) [23]. A meta-analysis of seven cohort studies revealed that a high TyG index significantly amplifies the risk of HTN development among the general population [24]. Although many cohort studies have explored the correlation between the TyG index and HTN, no studies have longitudinally investigated the link between the TyG index and the risk of progression to preHTN.

In a cross-sectional study of 108,370 adults without high FPG, Fan et al. [12] showed that the TyG index was not significantly associated with preHTN. Similarly, Zhen et al. [13] examined data from 105,070 nonobese adults (BMI, 18–24 kg/m^2^) and did not find an association between the TyG index and the risk of preHTN in men but noted such an association in women. Conversely, in a study of 3,274 Chinese adults without HTN or use of hypoglycemic or lipid-lowering medications, Zhanget al. [11] found a significantly higher proportion of individuals with preHTN in Q3 and Q4 of the TyG index than in Q1 (Q1 vs. Q3: odds ratio, 1.52 [95% CI, 1.20–1.92]; Q1 vs. Q4: odds ratio, 1.66 [95% CI, 1.31–2.61]), with no significant difference in Q2. Similarly, in a cross-sectional study involving 32,124 normoglycemic Chinese adults, Zhang et al. [9] reported a 1.091 times higher risk (95% CI 1.006–1.183) of preHTN among individuals in Q3 and a 1.795 times higher risk (95% CI 1.638–1.968) among those in Q4, with no significant difference in Q2. However, these studies demonstrating a significant relationship between the TyG index and preHTN risk did not conduct a sex-stratified analysis, leaving the relationship between the TyG index and preHTN in men and women unclear.

In the present 5-year longitudinal study conducted among the Japanese population, logistic regression analysis revealed results consistent with previous findings: individuals in Q3 and Q4 faced significantly high risks of progressing to preHTN compared with those in Q1 (Q1 vs. Q3: HR, 1.14 [95% CI, 1.02–1.30]; Q1 vs. Q4: HR, 1.28 [95% CI, 1.11–1.50]). Furthermore, our stratified analyses by sex, age, and BMI demonstrated that the risk of progressing to preHTN remained higher in Q3 and Q4 than in Q1 across these categories. Notably, factors such as male sex, advanced age, and obesity are recognized as pivotal factors for high BP. Consequently, the observed direct association between the TyG index and the risk of progression to preHTN may be confounded in individuals possessing these characteristics. Conversely, among low-risk populations, such as women, younger age, and nonobese individuals, a more distinct direct relationship between the TyG index and preHTN risk may emerge because of diminished confounding. The present stratified analyses by sex, age, and BMI indicated that even within these low-risk groups, higher TyG index quartiles corresponded to the increased risk of progression to preHTN. Therefore, the findings of the stratified analyses strongly support the conclusion that a high TyG index represents a direct risk factor for progression to preHTN among the Japanese population.

In a cross-sectional study of the Chinese population aged ≥ 65 years, Yaxin et al. [25] reported a discriminative ability for HTN with an AUC of 0.58 (95% CI, 0.56–0.60), with the optimal TyG index cutoff of 8.38. Similarly, another study involving US adults aged > 18 years reported an AUC for predicting HTN of 0.58 (95% CI, 0.57–0.59), with the optimal TyG index cutoff of 8.57 [26]. Furthermore, a cross-sectional study involving 105,070 lean Chinese adults without HTN revealed that the TyG index lacked sufficient discriminative performance to predict preHTN, yielding an AUC of 0.605 (95% CI, 0.600–0.611) [13]. Chen et al. [27] also reported an AUC of 0.632 (95% CI, 0.630–0.634) for predicting preHTN in Chinese individuals, with a TyG index cutoff of 8.4. Moreover, in the Japanese population, they reported an AUC of 0.676 (95% CI, 0.668–0.683), with a corresponding TyG index cutoff of 8.1 [27]. Consistent with these previous findings in cross-sectional studies, the present longitudinal cohort study demonstrates that the TyG index lacks sufficient discriminative performance to predict preHTN development, with an AUC of 0.600 (< 0.7) and a TyG index cutoff of 8.0. Consequently, relying solely on the TyG index may not be adequate for predicting preHTN development. A more effective strategy may involve combining the TyG index with other indicators such as BMI, WC, and WC to height ratio [13].

This study adds to the growing evidence of the correlation between insulin resistance and the risk of progression to preHTN. The principal strengths of this study lie in its longitudinal design, community-based cohort approach, and inclusion of a relatively large sample who were not taking TG-lowering drugs. However, this study has several limitations. First, the study design was retrospective and observational, which inherently prevents the establishment of definitive causal relationships. Second, serum sample variables were measured only once, although TG and FPG levels are known to fluctuate, potentially influenced by daily dietary intake and physical activity [28, 29]. Third, this study was confined to the Japanese population. Consequently, to assess the generalizability of the present findings to other ethnicities and populations, replication studies in additional cohorts are imperative. Furthermore, systemic inflammation was not evaluated in this study. Inflammation serves as a pivotal factor exacerbating insulin resistance and arterial HTN [30], potentially influencing the study outcomes.

## Conclusions

Herein, a higher TyG index was found to significantly correlate with the risk of progression to preHTN in a Japanese population, even after adjusting for conventional risk factors of HTN. The findings of this study indicate the clinical significance of the TyG index in refining early preHTN management. In this study, the dose–response curve showed a clear positive linear correlation between the TyG index and the risk of progression to preHTN, with the risk considerably increasing when the TyG index was > 8. Prospective studies are required to determine whether maintaining a TyG index of ≤ 8 can prevent progression to preHTN.

## Data Availability

The datasets used and/or analyzed during the current study are available from the corresponding author on reasonable request.

## Abbreviations

AUC: Area under the curve
BMI: Body mass index
BP: Blood pressure
CI: Confidence interval
DBP: Diastolic blood pressure
eGFR: Estimated glomerular filtration rate
FPG: Fasting blood glucose
HbA1c: Hemoglobin A1c
HDL-C: High-density lipoprotein cholesterol
HR: Hazard ratio
HTN: Hypertension
LDL-C: Low-density lipoprotein cholesterol
PreHTN: Prehypertension
Q: Quartile
ROC: Receiver operating characteristic
SBP: Systolic blood pressure
TC: Total cholesterol
TG: Triglyceride
TyG: Triglyceride-glucose
UA: Uric acid
WC: Waist circumference
